# Whitefly Species Preferences of the Predatory Ladybird Beetle, *Delphastus pallidus* LeConte (Coleoptera: Coccinellidae)

**DOI:** 10.3390/insects17010090

**Published:** 2026-01-13

**Authors:** Muhammad Z. Ahmed, Catharine M. Mannion, Cindy L. McKenzie, Lance S. Osborne

**Affiliations:** 1 Pee Dee Research and Education Center, Clemson University, 2200 Pocket Road, Florence, SC 29506, USA; 2 Department of Entomology and Nematology, Tropical Research and Education Center, University of Florida–IFAS, 18905 SW 280th Street, Homestead, FL 33031, USA; 3 Subtropical Insects and Horticulture Research Unit, Agricultural Research Service, United States Department of Agriculture, 2001 South Rock Road, Fort Pierce, FL 34945, USA; 4 Department of Entomology and Nematology, Mid-Florida Research and Education Center, University of Florida–IFAS, 2725 South Binion Road, Apopka, FL 32703, USA; lsosborn@ufl.edu

**Keywords:** biological control, invasive whitefly species, ladybird beetle, ornamental landscape, predatory beetles

## Abstract

*Delphastus* Casey (Coleoptera: Coccinellidae: Serangiini) comprises small predatory ladybird beetles that feed on immature whiteflies. Several *Delphastus* species are utilized as biological control agents. However, *Delphastus pallidus* (LeConte) has been understudied for the past several decades. Recent landscape surveys in South Florida revealed a marked increase in *D. pallidus* populations associated with multiple whitefly species. We evaluated whitefly species preferences of *D. pallidus* using choice and no-choice assays conducted in incubators and insectaries. Seven whitefly species were confirmed as prey and ranked in order of preference: *Bemisia tabaci* Gennadius > *Aleurothrixus trachoides* (=*Aleurotrachelus trachoides*) (Back) > *Singhiella simplex* (Singh) > *Paraleyrodes bondari* Peracchi > *Aleurodicus rugioperculatus* Martin > *Asiothrixus antidesmae* (Takahashi) > *Aleurodicus dugesii* Cockerell. Findings indicate that *D. pallidus* feeds on a taxonomically and morphologically diverse set of whiteflies, including both waxy and non-waxy species, which likely facilitates establishment and dispersal in ornamental and agricultural landscapes. This study is the first to document *D. pallidus* as a potentially native U.S. predatory beetle that attacks at least seven whitefly species. Knowledge of its prey preferences will inform mass-rearing strategies and potential deployment for whitefly biocontrol.

## Simple Summary

*Delphastus* is a genus of small coccinellid beetles that specialize in whiteflies. Recent surveys have documented an increased abundance of the potentially native United States species *Delphastus pallidus*, which feeds on multiple whitefly species in South Florida. In controlled preference trials, we identified seven whitefly species as suitable prey. *Bemisia tabaci* was the most strongly preferred. These results show that *D. pallidus* accepts both waxy and non-waxy whiteflies, indicating its promise as a biological control agent for a range of whitefly pests.

## 1. Introduction

*Delphastus* Casey (Coleoptera: Coccinellidae: Serangiini) comprises small predatory ladybird beetles that prey on whiteflies. These beetles attack immature stages of whiteflies and are commercially reared and distributed globally for whitefly control [1]. Notably, many *Delphastus* species are compatible with parasitoids because adults and larvae tend to avoid parasitized whitefly nymphs, reducing intraguild conflict in integrated pest management programs [2]. Within the genus, several species are recognized as biological control agents, including *Delphastus catalinae* (Horn) [previously *D. pusillus* (LeConte)], *D. davidsoni* (Gordon), and *D. pallidus* (LeConte). However, over the past seven decades, there has been relatively little published information on *D. pallidus*, a species reported from the United States [1,3,4,5]. Most studies have focused on *D. catalinae*, which was previously misidentified in the literature as *D. pusillus*, because *D. catalinae* is more readily available commercially [6].

The earliest documented prey records of *D. pallidus* in Florida date to the early 1950s, when it was observed feeding on the citrus whitefly, *Dialeurodes citri* (Ashmead), in Vero Beach and the Mims area in 1951 and later in Indian Rocks and Lake Alfred in 1953 [3]. Subsequent records include a specimen with the locality label “Sand Point, Florida” (city and county not specified), as well as additional museum and survey specimens that extend the known distribution of *D. pallidus* to other US localities and several countries in the Caribbean, South America, and Asia [4,5]. Reported occurrences outside the continental US include Hawaii, Ecuador, the Bahamas, Cuba, the Dominican Republic, the Virgin Islands, and Pakistan [4,5,7].

Historically, *D. pallidus* populations in Florida agroecosystems have been reported as sparse, with records from eight counties, primarily in southern Florida: Brevard, Broward, Charlotte, Martin, Miami-Dade, Monroe, Palm Beach, and Sarasota [1,8]. They were recorded among predators of *Aleurodicus rugioperculatus* Martin (2012–2014) and subsequently observed feeding on *Aleurothrixus trachoides* (Back) (formerly *Aleurotrachelus trachoides* Back) in 2015 [9,10]. However, recent surveys have documented a notable and consistent increase in *D. pallidus* on *Ficus benjamina* L. hedges infested with ficus whitefly, *Singhiella simplex* (Singh), across multiple locations in Miami-Dade County, Florida [1,8]. Concurrent surveys to assess host range revealed *D. pallidus* feeding on at least four additional whitefly species in the region: *Bemisia tabaci* Gennadius (sweetpotato/silverleaf whitefly; hereafter *B. tabaci*), *A. trachoides* (pepper/solanum whitefly), *Paraleyrodes bondari* Peracchi (Bondar’s nesting whitefly; BNW), and *Dialeurodes citrifolii* Morgan (cloudy-winged whitefly) [1].

Field observations indicate that several whitefly species commonly occur on different host plants growing side-by-side in ornamental landscapes. This spatial proximity creates opportunities for mobile predators to move among host plants and prey species. For example, *B. tabaci* and *Aleurodicus* spp. were observed on *Hibiscus rosa-sinensis* L.; *Asiothrixus antidesmae* (Takahashi) on *Ixora* sp.; *S. simplex* and *P. bondari* on *F. benjamina*; *A. trachoides* on *Duranta erecta* L. and *Capsicum annuum* L.; and *A. rugioperculatus* on *Strelitzia reginae* Banks [8,10]. These spatially proximate host–prey assemblages create opportunities for a mobile predator to move among whitefly species and host plants, thereby influencing local predator distribution and prey attack rates [1,8].

Based on our surveys and landscape observations, we identified at least seven whitefly species commonly found in proximity (*B. tabaci*, *A. trachoides*, *S. simplex*, *P. bondari*, *A. dugesii*, *A. antidesmae*, and *A. rugioperculatus*) and therefore evaluated the whitefly species preference of *D. pallidus* against these seven species [1,8]. Understanding prey preference will inform which species are most suitable as hosts for future mass-rearing, whether *D. pallidus* can be deployed against multiple whitefly pests, and whether a release targeting one species is likely to result in movement to and suppression of other whitefly species on nearby plants.

## 2. Materials and Methods

### 2.1. Whitefly Colony Maintenance

Colonies of seven whitefly taxa—*B. tabaci* (SPW), *A. trachoides* (PW), *S. simplex* (FW), *P. bondari* (BNW), *A. dugesii* (GW), *A. antidesmae* (IW), and *A. rugioperculatus* (RSW)—were maintained on their field-associated host plants in greenhouse conditions (18–24 °C, 65–80% RH) for several months prior to experiments [1,8]. Freshly infested plants were rotated regularly to sustain colonies.

### 2.2. Whitefly Identifications

Pseudopupae (fourth instars) were slide-mounted following the technique in [11] and identified using the key in [11] upon colony establishment and quarterly thereafter to verify species identity and colony purity. For taxa not covered in [11], we used the key in [8] for *P. bondari*, *S. simplex*, and *A. rugioperculatus*, and the original species description for *A. antidesmae* from [12]. Voucher slides were archived for traceability.

### 2.3. Beetle Colony Maintenance

Adult *D. pallidus* were collected from BNW- and FW-infested *F. benjamina* hedges with an aspirator (BioQuip model 2809TS). The beetles were then transferred to plant cages (37 × 37 × 60 cm) containing potted *F. benjamina* infested with BNW and FW. Cages were maintained at 25 ± 1 °C, 65 ± 5% RH, 13:11 L:D. Each month, a fresh infested *F. benjamina* plant (2-gal pot) was added to the cage, and older plants were replaced to maintain colony continuity. Only adults 24–72 h post-eclosion were used in experiments. Beetles were starved for 24 h prior to release to standardize hunger. Beetle age cohorts, holding transfers, and colony manipulations were recorded in experiment logs. Any beetle removed for dissection or damaged during handling was replaced from a holding cohort, and all replacement events were logged.

### 2.4. Defining and Quantifying Accessible Immatures

“Accessible immatures” were defined as eggs plus nymphal instars visually exposed on the abaxial leaf surface and reachable without mechanically removing wax or other coverings. Candidate leaf sections were screened under a dissecting stereo microscope (10–40×). Sections were adjusted to target ≈50 accessible immatures per Petri dish leaf and ≈200 accessible immatures per branch for box assays.

### 2.5. Experimental Arena and Unit

Petri dish choice assays used 150 mm plastic dishes lined with filter paper, placed over a water-filled cup reservoir (p-cup arrangement) to maintain leaf turgor and local humidity (Figure S1). Detached leaf sections bearing target accessible immatures were mounted. A damp cotton ball provided humidity. Replication: five dishes per treatment per run × three independent runs = 15 independent dish arenas per treatment. The dish arena was the experimental unit, and all primary inferential tests were conducted on arena-level summaries (mean beetle occupancy per arena).

Box (macro-arena) choice assays used glass-topped boxes (4 × 3 × 1 ft) containing seven branches (one branch per species) with petioles in 225 mL flasks. Each branch targeted ≈200 accessible immatures. This use of branches placed in flasks provided a convenient and standardized method to approximate natural host presentation, although it does not fully replicate intact plant architecture. The beetles were released centrally at the base of the box arena, allowing equal access to all flasks. Movement to host foliage included both crawling along flask surfaces and flying within the arena. Replication: three arenas per replication × three replications = 9 independent box arenas. The box arena was the experimental unit for box assays.

### 2.6. Beetle Allocation and Replacement Procedures

To equalize initial per-prey beetle density in multi-prey Petri dishes, five adult beetles were released per prey species per dish (e.g., a seven-species dish received 35 beetles). No-choice controls were used. Dead beetles observed during the experimental interval were immediately replaced from a separate holding cohort and logged in both the Petri dish and box-arena assays. Arenas with cumulative beetle mortality > 20% during the experiment were flagged. These arenas were excluded from the ANOVAs. All replacement events, causes, and timestamps were recorded in experiment logs.

### 2.7. Randomization and Scoring Procedures

Arena positions within growth chambers and the insectary were assigned by random number tables and rotated daily to minimize positional and microclimate bias. Beetles were drawn at random from holding cohorts, and observers were not given beetle or replicate identities. Observers recorded the number of adult beetles present on each infested leaf every 15 min during a 6 h daily observation window (08:00–14:00) until leaf turgor prevented reliable counting. Repeated counts were aggregated to an arena mean per observation day and then to the overall arena mean across days for primary analyses.

Counts were performed visually under controlled lighting using a dissecting microscope or magnifier as needed. Each run was scored by a single trained observer to ensure consistency. Observers logged any uncertain counts, along with contextual notes, and these annotations were retained in the experiment logs to ensure traceability.

### 2.8. Statistical Design and Analysis

For transparency, we report two complementary analysis levels. First, pooled observation-level ANOVAs (tests on repeated 15 min counts aggregated across the observation window) are presented in figure panels to show temporal replication. The pooled F, df, and *p*-values are reported in figure panels and captions. Second, arena-level summaries (one mean per independent Petri-dish or box arena; arena = experimental unit; n = 15 Petri arenas per treatment; n = 18 box arenas per treatment) were computed and used as confirmatory sensitivity checks; arena-level summaries reproduced the ordinal prey ranking and did not change principal conclusions. Arena-level ANOVAs on means (arena = experimental unit) were run as confirmatory tests; where pooled and arena-level outcomes differ materially, both results are presented and discussed. Residuals were inspected for normality (Shapiro–Wilk) and homogeneity of variance (Levene). When diagnostics indicated departures from ANOVA assumptions, square-root or log transformations were applied. The specific transformation used for each test is stated in Section 3 and in the corresponding figure caption. Arenas with cumulative beetle mortality >20% were flagged and excluded from arena-level ANOVAs (report count Y in Results and captions). All ANOVAs and post hoc tests (Tukey’s HSD) were implemented in SAS 9.4. Figures display arena means ± SE. Pooled-analysis df and F values are shown in figure panels and captions.

Raw experiment logs (counts, replacement/mortality notes, and accessible immature tallies) were maintained for internal record-keeping. Results and figures in the manuscript use aggregated arena-level summaries as described. Sensitivity re-analyses that retain flagged arenas are summarized in the Results to demonstrate the robustness of the primary inferences. Figures and Results state sample sizes used for each test and present arena means ± SE.

## 3. Results

### 3.1. Petri Dish Experiment 1: PW vs. FW

Adult *D. pallidus* manifested a robust and reproducible preference for PW (*A. trachoides*) relative to FW (*S. simplex*). Mean beetle occupancy was 1.52 ± 0.10 adults per arena on FW and 2.78 ± 0.14 adults per arena on PW. These means were calculated across the 50 daily aggregated observations per arena used in pooled tests (pooled observation-level ANOVA: F_1,98_ = 213.34, *p* < 0.001). Residual diagnostics indicated a modest positive skew in the raw counts. Applying a square root transformation corrected heteroscedasticity and met model assumptions without altering the direction or significance of the effect. An arena-level ANOVA on dish means (arena = Petri dish; n = 15) corroborated the pooled inference.

### 3.2. Petri Dish Experiment 2: FW vs. BNW

**Figure 1:**
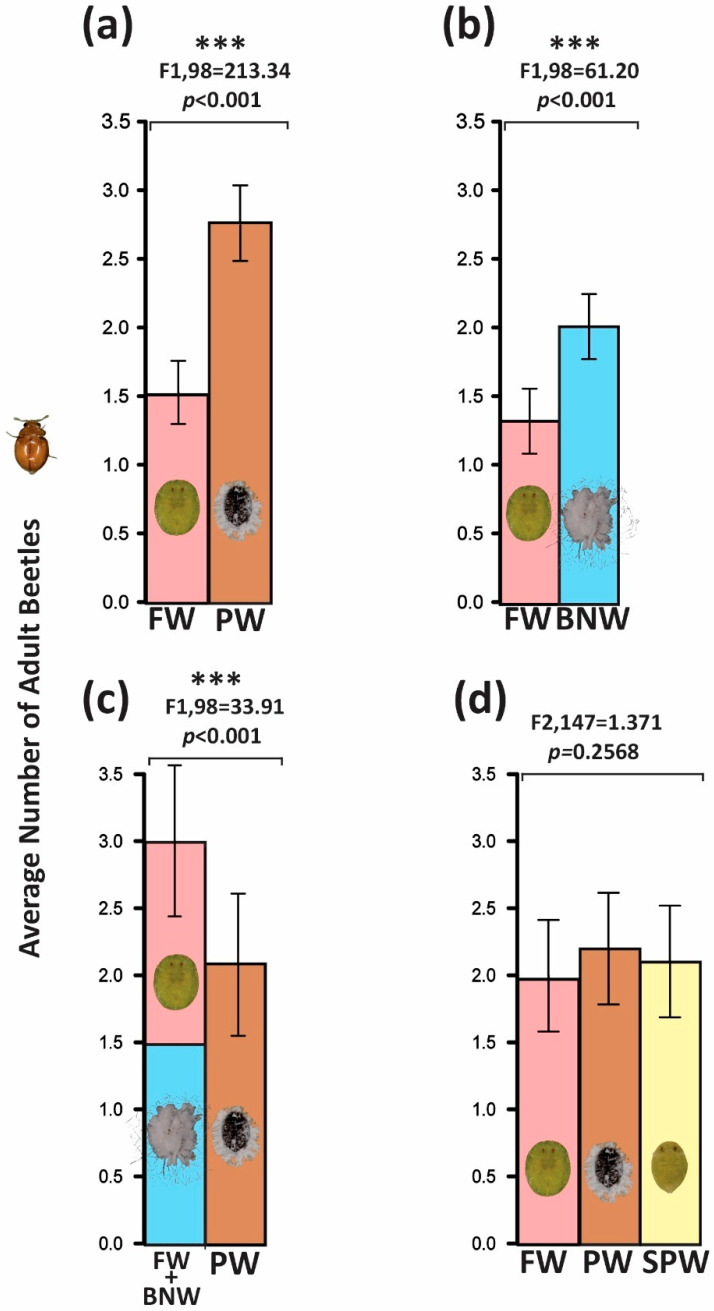
(**a**) Binary choice assay quantifying relative selection by *Delphastus pallidus* between Ficus whitefly (FW), *Singhiella simplex*, and pepper whitefly (PW), *Aleurothrixus trachoides* (=*Aleurotrachelus trachoides*); mean occupancy and sustained residence on PW exceeded those on FW (FW 1.52 ± 0.10; PW 2.78 ± 0.14; pooled observation-level ANOVA: F_1,98_ = 213.34, *p* < 0.001; square-root transformation applied; arena-level ANOVA on means (n = 15) confirmed the result). (**b**) Binary choice assay between FW and Bondar’s nesting whitefly (BNW), *Paraleyrodes bondari*, showing consistent BNW preference (FW 1.26 ± 0.09; BNW 2.00 ± 0.13; pooled ANOVA: F_1,98_ = 61.20, *p* < 0.001; log transformation applied where indicated by diagnostics; arena-level ANOVA on dish means (n = 15) confirmed the result). (**c**) Comparative allocation to a heterogeneous patch (BNW + FW) versus PW alone, indicating greater attendance to the mixed assemblage (PW 2.12 ± 0.13; BNW + FW 2.99 ± 0.17; pooled ANOVA: F_1,98_ = 33.91, *p* < 0.001; transformation details are reported in the Results; arena-level ANOVA on dish means (n = 15) confirmed the result). (**d**) Ternary choice among PW, FW, and sweetpotato whitefly (SPW), *Bemisia tabaci* (B biotype/MEAM1), in which mean beetle counts did not differ significantly (PW 2.21 ± 0.49; FW 1.98 ± 0.49; SPW 2.11 ± 0.48; pooled ANOVA: F_2,147_ = 1.371, *p* = 0.2568; no transformation required; arena-level ANOVA on dish means (n = 15) confirmed the non-significant outcome). Three asterisks (*) indicate a highly significant difference.

When FW (*S. simplex*) and BNW (*P. bondari*) were juxtaposed on *F. benjamina* under identical arena conditions, *D. pallidus* consistently selected BNW (pooled ANOVA: F_1,98_ = 61.20, *p* < 0.001) (Figure 1b). Mean beetle occupancy averaged 1.26 ± 0.09 adults per arena on FW and 2.00 ± 0.13 adults per arena on BNW. These means were calculated across the 50 daily aggregated observations per arena used in pooled tests. A log transformation was applied where required by diagnostics, and an arena-level ANOVA on dish means (arena = experimental unit) corroborated the pooled outcome. The effect was consistent across the three independent runs and persisted after log transformation, where required to satisfy the ANOVA assumptions, suggesting that prey-intrinsic cues, rather than host-leaf artifacts, drive colonization.

### 3.3. Petri Dish Experiment 3: BNW + FW vs. PW

In comparison of a composite BNW (*P. bondari*) + FW (*S. simplex*) patch versus PW (*A. trachoides*) alone, beetles showed significantly greater attendance to the mixed BNW + FW treatment (pooled ANOVA: F_1,98_ = 33.91, *p* < 0.001) (Figure 1c). Mean beetle occupancy averaged 2.12 ± 0.13 adults per arena on PW and 2.99 ± 0.17 adults per arena on the mixed BNW + FW patch. The means were calculated across the 50 daily aggregated observations per arena used in pooled tests. An arena-level ANOVA on dish means (arena = experimental unit) corroborated the pooled outcome. This preferential allocation to heterogeneous patches is consistent with either additive attraction to composite prey cues or higher accessibility to adequate prey in mixed assemblages. Randomized dish placement and rotational scheduling (see Section 2) rule out positional confounds.

### 3.4. Petri Dish Experiment 4: FW, PW, and SPW

A triadic choice among FW (*S. simplex*), PW (*A. trachoides*), and SPW (*B. tabaci*) on their respective host leaves produced no statistically significant differences in mean beetle counts (pooled ANOVA: F_2,147_ = 1.371, *p* = 0.2568) (Figure 1d). Mean occupancy averaged 2.21 ± 0.49 adults per arena on PW, 1.98 ± 0.49 adults per arena on FW, and 2.11 ± 0.48 adults per arena on SPW. The means were calculated across the 50 daily aggregated observations per arena used in pooled tests. No transformation was required for this comparison. An arena-level ANOVA on dish means (arena = experimental unit; n = 15) corroborated the pooled non-significant outcome. Under rigorously controlled immature densities and standardized leaf conditions, *D. pallidus* showed functional equivalence in acceptance among these three taxa, indicating facultative generalism among less heavily waxed or readily accessible prey.

### 3.5. Petri Dish Experiment 5: BNW, FW, GW, PW, RSW, and SPW

**Figure 2:**
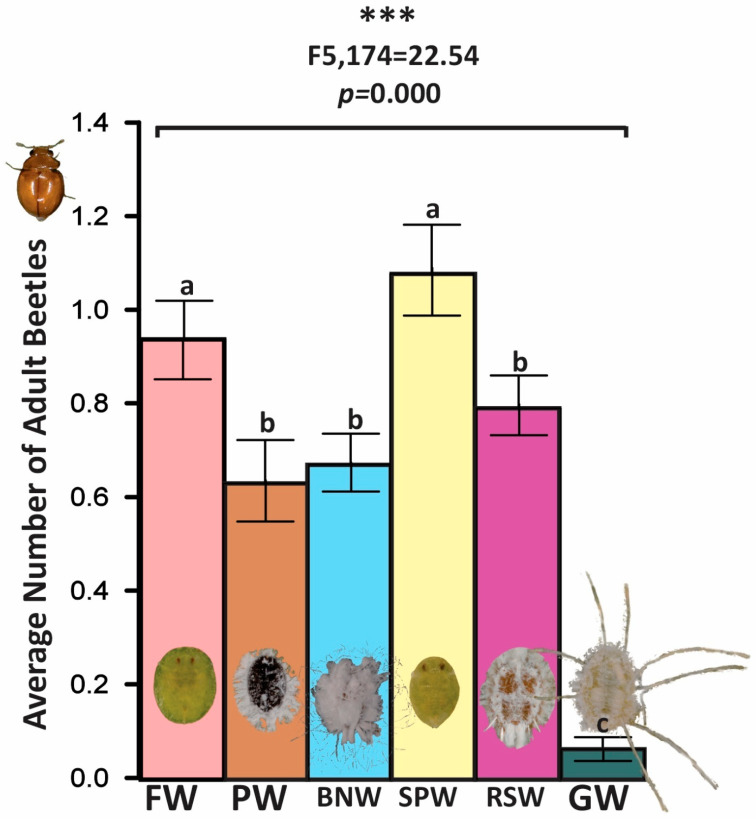
Multi-choice assay presenting prey-preference indices for *Delphastus pallidus* across six whitefly taxa with standardized accessible immature availability: Bondar’s nesting whitefly (BNW), *Paraleyrodes bondari*; Ficus whitefly (FW), *Singhiella simplex*; giant whitefly (GW), *Aleurodicus dugesii*; pepper whitefly (PW), *Aleurothrixus trachoides* (=*Aleurotrachelus trachoides*); rugose spiraling whitefly (RSW), *Aleurodicus rugioperculatus*; and sweetpotato whitefly (SPW), *Bemisia tabaci* (B biotype/MEAM1). Overall occupancy differed among prey (PW 0.63 ± 0.09; BNW 0.67 ± 0.06; RSW 0.79 ± 0.07; FW 0.94 ± 0.08; SPW 1.08 ± 0.10; GW 0.07 ± 0.02; pooled ANOVA: F_5,174_ = 22.54, *p* < 0.001; arena-level ANOVA on dish means (n = 15) confirmed the outcome); FW and SPW formed an upper preference tier, BNW/PW/RSW an intermediate tier, and GW the lowest occupancy, a pattern attributable to differential availability of immature prey and impediments caused by heavy wax coverings. Three asterisks (*) indicate a highly significant difference. Different letters show significant differences based on the LSD value.

In six-prey arenas that standardized accessible immature availability when feasible (BNW [*P. bondari*], FW [*S. simplex*], GW [*A. dugesii*], PW [*A. trachoides*], RSW [*A. rugioperculatus*], SPW [*B. tabaci*]), overall occupancy differed significantly among prey types (pooled ANOVA: F_5,174_ = 22.54, *p* < 0.001) (Figure 2). An arena-level ANOVA on dish means (n = 15) corroborated the pooled significant outcome. FW (*S. simplex*) and SPW (*B. tabaci*) formed an upper preference tier with the greatest beetle densities. BNW (*P. bondari*), PW (*A. trachoides*), and RSW (*A. rugioperculatus*) constituted an intermediate tier with statistically indistinguishable occupancies. GW (*A. dugesii*), characterized by extensive wax coverings and reduced accessible-immature counts, sustained the fewest beetles. Mean occupancy averaged PW 0.63 ± 0.09, BNW 0.67 ± 0.06, RSW 0.79 ± 0.07, FW 0.94 ± 0.08, SPW 1.08 ± 0.10, and GW 0.07 ± 0.02 adults per arena. The means were calculated across the 30 daily aggregated observations per arena. Sensitivity analyses that excluded replicates in which GW accessible counts deviated from the target confirmed the same ordinal pattern.

### 3.6. Box Experiment: BNW, FW, GW, PW, RSW, SPW, and IW

**Figure 3:**
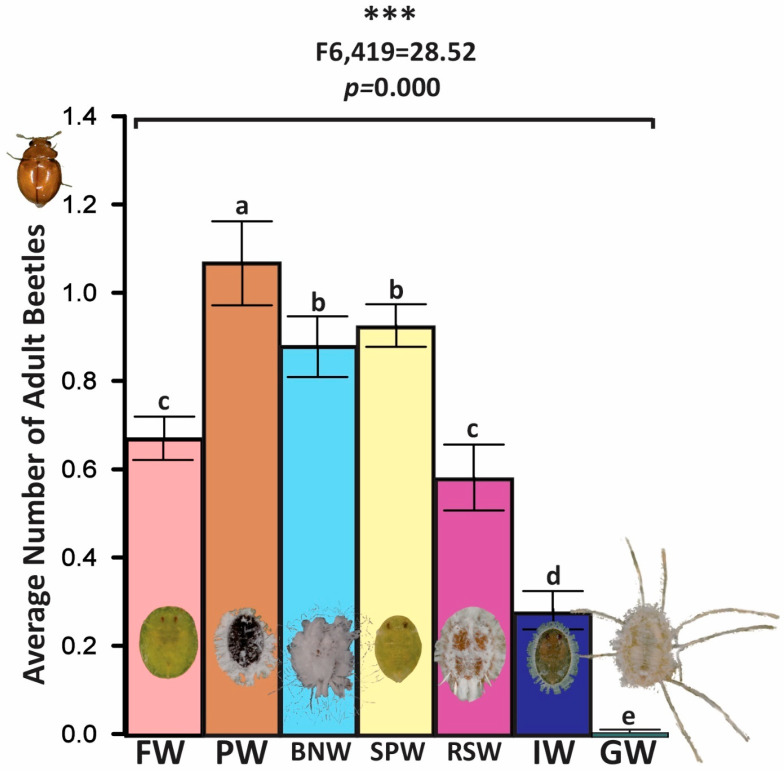
Spatially explicit box assay reporting relative beetle densities among seven sympatric whitefly taxa presented on their field-associated host branches (~200 accessible immatures per branch; randomized flask arrangement). Distribution differed significantly among prey (PW 1.07 ± 0.12; BNW 0.88 ± 0.10; RSW 0.72 ± 0.08; FW 0.67 ± 0.05; SPW 0.93 ± 0.05; IW 0.28 ± 0.05; GW 0.01 ± 0.01; pooled ANOVA: F_6,419_ = 28.52, *p* < 0.001; arena-level ANOVA on dish means (n = 18) confirmed the outcome); *Aleurothrixus trachoides* (PW) attracted the highest densities, followed by *Paraleyrodes bondari* (BNW) and *Bemisia tabaci* (SPW), which were statistically equivalent; *Singhiella simplex* (FW) and *Aleurodicus rugioperculatus* (RSW) occupied an intermediate rank; *Asiothrixus antidesmae* (IW) was ranked next, and *Aleurodicus dugesii* (GW) had the lowest densities. These results corroborated patterns from Petri dish trials while revealing movement dynamics and patch-selection behavior at a larger spatial scale. Three asterisks (*) indicate a highly significant difference. Different letters show significant differences based on the LSD value.

In spatially expansive box assays presenting seven species concurrently on their field-associated hosts (≈200 accessible immatures per branch; randomized flask arrangement), beetle distribution varied significantly among prey (pooled ANOVA: F_6,419_ = 28.52, *p* < 0.001) (Figure 3). PW (*A. trachoides*) attracted the highest beetle densities, followed by BNW (*P. bondari*) and SPW (*B. tabaci*), which were statistically equivalent. FW (*S. simplex*) and RSW (*A. rugioperculatus*) formed an intermediate tier; IW (*A. antidesmae*) was ranked next, and GW (*A. dugesii*) had the lowest attendance. Mean occupancy was PW 1.07 ± 0.12, BNW 0.88 ± 0.10, RSW 0.72 ± 0.08, FW 0.67 ± 0.05, SPW 0.93 ± 0.05, IW 0.28 ± 0.05, and GW 0.01 ± 0.01 adults per arena. The means were calculated across the 60 daily aggregated observations per arena. An arena-level ANOVA on dish means (n = 18) corroborated the pooled outcome. These spatially explicit assays corroborated patterns from Petri dish trials, while also revealing movement dynamics and patch-selection behavior at a more naturalistic spatial scale.

## 4. Discussion

The present study provides the first taxonomically validated, simultaneous evaluation of prey preference in *Delphastus pallidus* across seven sympatric whitefly species. Our assays revealed a consistent ordinal ranking, with *Bemisia tabaci* most strongly preferred, followed by *Aleurothrixus trachoides*, *Singhiella simplex*, *Paraleyrodes bondari*, *Aleurodicus rugioperculatus*, *Asiothrixus antidesmae*, and *A. dugesii*. These findings demonstrate that *D. pallidus* readily accepts both wax-covered and non-waxy taxa, underscoring its potential as a broadly effective biological control agent in ornamental and agricultural landscapes. Within the broader assemblage of whitefly predators, the only predatory beetle previously documented across numerous whitefly taxa is *Nephaspis oculata* (Blatchley), with observational records implicating predation on ten whitefly species, including avocado whitefly (*Trialeurodes floridensis* Quaintance) [13], citrus blackfly (*Aleurocanthus woglumi* Ashby) [14], citrus whitefly (*Dialeurodes citri* Ashmead), cloudy-winged whitefly (*D. citrifolii* Morgan) [13], croton whitefly (*Orchamoplatus mammeferus* Quaintance & Baker) [15], hollyleaf cherry whitefly (*Pealius kelloggi* Bemis) [16,17], RSW [18], spiraling whitefly (*A. dispersus* Russell) [19], SPW (B biotype/MEAM1) [20], and woolly whitefly (*A. floccosus* Maskell) [15]. These historical accounts come from geographically disparate studies, few of which combined concurrent laboratory tests across multiple prey species with taxonomic verification. By contrast, the present study constitutes the first simultaneous, taxonomically validated laboratory evaluation of preference among seven sympatric whitefly species, enabled by exhaustive landscape surveys, rigorous taxonomic and colony authentication using established keys and voucher slides [8,11,12].

Within the broader assemblage of potential whitefly predators (≥30 species) identified in prior syntheses, several coccinellids (including *D. catalinae* and *D. pallidus*) and non-coccinellid predators are implicated in whitefly suppression. *N. oculata* remains the most broadly documented multi-prey beetle, but it is introduced in many regions [17,20,21,22]. Our experiments demonstrate that *D. pallidus*, a species with historical records on *D. citri* and *D. citrifolii* [1,3] and recent observations on *A. dispersus* in Hawaii [5,7,8], consistently accepts seven whitefly species under laboratory conditions, positioning it among the limited set of beetles with verified multi-prey utility in temperate and subtropical landscapes.

Two proximate determinants emerged as primary drivers of the observed prey ranking. First, accessible immature availability (eggs plus exposed nymphs) correlated positively with beetle attendance in confined arenas: taxa offering greater accessible immatures (for example, *B. tabaci*, *S. simplex*) supported higher mean beetle counts. Second, prey morphology, principally wax production, constrained exploitation; heavily waxed taxa (notably *A. dugesii*) registered the fewest beetles. This pattern was consistent with increased handling time and reduced attack efficiency. Mixed patch presentations (e.g., *P. bondari* + *S. simplex*) elicited additive attendance compared with single-prey patches. This pattern suggests that composite volatile or contact cues increase local foraging profitability in heterogeneous assemblages. Assay scale and arena configuration influenced behavioral expression. Petri dish arenas were selected to permit strict control of prey availability, leaf turgor, and incubator microclimate—critical when working with beetles.

The applied implications are immediate. The consistent acceptance of *B. tabaci* and its relative ease of rearing on Hibiscus recommend *B. tabaci* as a pragmatic host for mass production of *D. pallidus*. Prior work demonstrating marked oviphagy by *D. catalinae* and *D. pallidus*—consumption > 50 eggs d^−1^ and preference for eggs over nymphs, with no differential predation across two *B. tabaci* cryptic species—further supports *B. tabaci* as an efficient production host [23]. Field corroboration of high *D. pallidus* abundance on *B. tabaci* in other regions adds empirical weight to this recommendation [7]. Release strategies that target abundant, easily reared hosts are likely to produce both direct suppression of the focal whitefly species, as well as incidental suppression of proximate whitefly taxa because *D. pallidus* relocates among co-occurring hosts, as demonstrated in box assays. Integration with conservation and augmentative biocontrol tactics will require explicit attention to release timing, local host assemblages (including *P. bondari* and *S. simplex*), and plant spatial configuration to optimize encounter rates and retention.

To translate mechanistic insight into operational practice, we recommend three research trajectories. First, greenhouse and semi-field experiments should quantify realized prey suppression and movement dynamics across mixed host assemblages under realistic microclimatic regimes. Second, rigorous functional response assays should estimate handling time, attack rate, and per capita consumption for waxy versus non-waxy taxa to support population-level suppression models and evidence-based release densities. Third, controlled interaction trials with co-occurring natural enemies—particularly whitefly parasitoids—are necessary to assess intraguild compatibility and to design integration strategies that avoid antagonistic outcomes. These steps will position *D. pallidus* in a direct comparative context with other generalist predators and introduced multi-prey beetles, such as *N. oculata* [13,14,15,16,17,18,19,20,21,23].

## 5. Conclusions

By integrating Petri dish and box assays, we demonstrate that *D. pallidus* feeds on seven sympatric whitefly species and expresses a reproducible prey ranking (SPW > PW > FW > BNW > RSW > IW > GW) under the experimental conditions described. The beetle accepts both wax-covered and non-waxy taxa. It responds to the availability of immature hosts and can relocate among proximate hosts. These traits support its candidacy as a flexible biological-control agent. Realizing this potential will require semi-field and field validation of per-capita predation efficacy, optimization of mass-rearing protocols (with SPW/*B. tabaci* as a leading host), and carefully designed integration trials with existing natural enemies before operational deployment.

## Data Availability

The raw data used for the analyses are openly available in ‘Mendeley data.’ https://doi.org/10.17632/dy6vt54zz8.1.

## Supplementary Materials

The following supporting information can be downloaded at: https://www.mdpi.com/article/10.3390/insects17010090/s1, Figure S1: (A) Petri dish p-cup arenas used to assay prey selection by the predatory coccinellid *Delphastus pallidus*: detached host leaves bearing standardized densities (~50 accessible immatures per leaf) were presented concurrently, and beetle occupancy was recorded. (B) Macro-arena box configuration conducted on plants: a ventilated box containing flasks with infested foliage arranged to permit movement of *D. pallidus* among distinct whitefly-bearing substrates.
