# Supplementary material for: Whitefly Species Preferences of the Predatory Ladybird Beetle, Delphastus pallidus LeConte (Coleoptera: Coccinellidae)
Source: Insects. 2026 Jan 13;17(1):90. doi: 10.3390/insects17010090 (PMC12841646; doi:10.3390/insects17010090)
Supplement: Supplementary file 1 [file insects-17-00090-s001.zip › insects-3932514-supplementary.pdf]

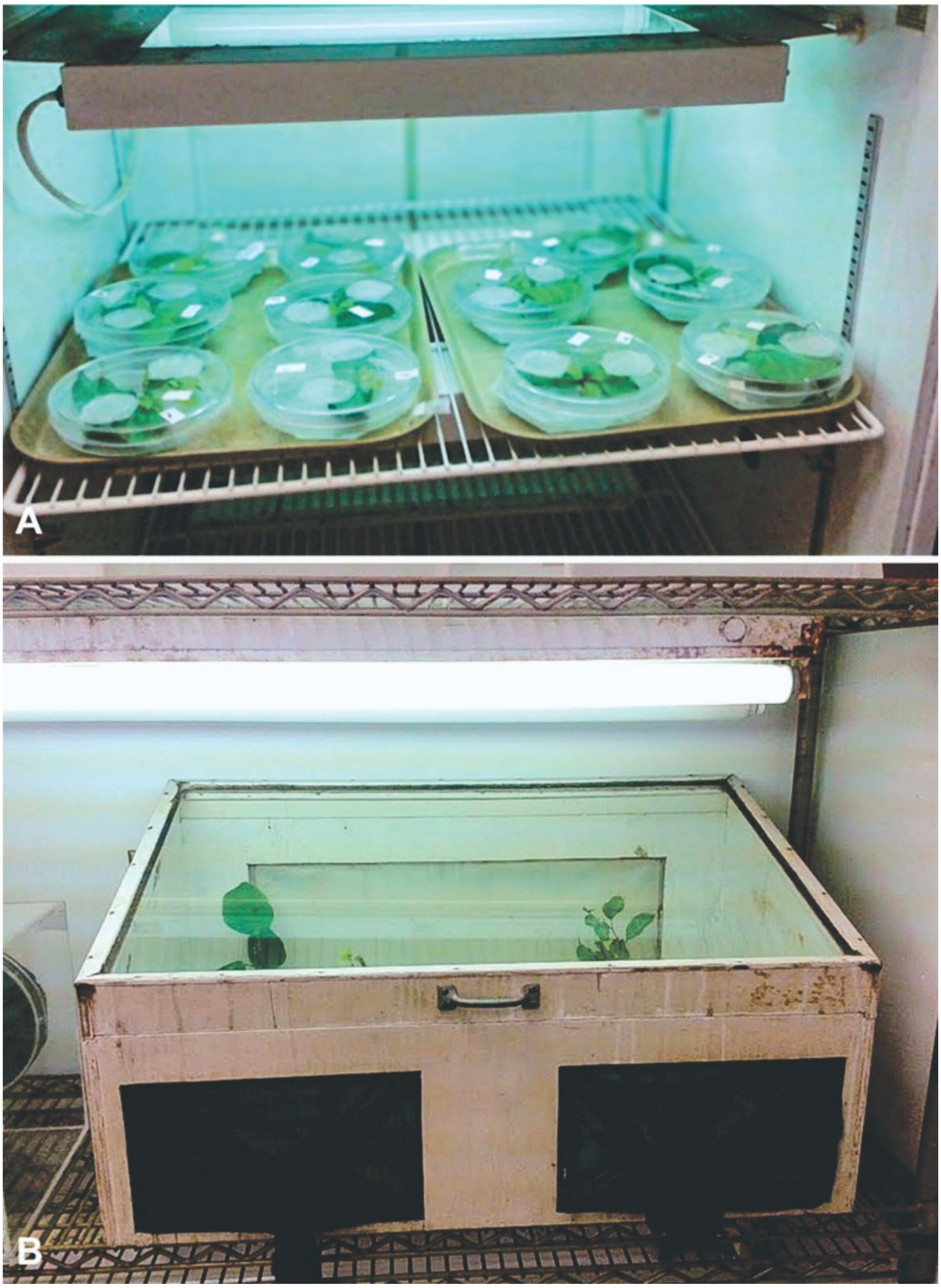

Figure S1. (A) Petri dish p-cup arenas used to assay prey selection by the predatory coccinellid *Delphastus pallidus*: detached host leaves bearing standardized densities (~50 accessible immatures per leaf) were presented concurrently, and beetle occupancy was recorded. (B) Macro-arena box configuration conducted on plants: a ventilated box containing flasks with infested foliage arranged to permit movement of *D. pallidus* among distinct whitefly-bearing substrates.
